# Correlation between ABO Blood Group Phenotype and the Risk of COVID-19 Infection and Severity of Disease in a Saudi Arabian Cohort

**DOI:** 10.1007/s44197-021-00023-3

**Published:** 2022-01-03

**Authors:** Dunia Jawdat, Ali Hajeer, Salam Massadeh, Nora Aljawini, Malak S. Abedalthagafi, Manal Alaamery

**Affiliations:** 1 Saudi Stem Cells Donor Registry, King Abdullah International Medical Research Center, King Saud Bin Abdulaziz University for Health Sciences, King Abdulaziz Medical City, Ministry of National Guard Health Affairs, Riyadh, Saudi Arabia; 2 Department of Pathology and Laboratory Medicine, Ministry of National Guard Health Affairs, King Abdullah International Medical Research Center/ King Saud bin Abdulaziz University for Health Sciences, Riyadh, Saudi Arabia; 3 Developmental Medicine Department, King Abdullah International Medical Research Center, King Saud Bin Abdulaziz University for Health Sciences, King Abdulaziz Medical City, Ministry of National Guard- Health Affairs, P.O Box 22490, Riyadh, 11426 Saudi Arabia; 4 KACST-BWH Centre of Excellence for Biomedicine, Joint Centers of Excellence Program, King Abdulaziz City for Science and Technology (KACST), Riyadh, Saudi Arabia; 5 King Abdulaziz City for Science and Technology (KACST)-Saudi Human Genome Satellite Lab at King Abdulaziz Medical City, Ministry of National Guard Health Affairs (MNGHA), Riyadh, Saudi Arabia; 6 Genomics Research Department, King Fahad Medical City and King Abdulaziz City for Science and Technology, Riyadh, Saudi Arabia

**Keywords:** COVID-19, SARS-CoV-2, ABO, Risk, Saudi Arabia

## Abstract

**Background:**

Disease severity among patients infected with SARS-CoV-2 varies remarkably. Preliminary studies reported that the ABO blood group system confers differential viral susceptibility and disease severity caused by SARS-CoV-2. Thus, differences in ABO blood group phenotypes may partly explain the observed heterogeneity in COVID-19 severity patterns, and could help identify individuals at increased risk. Herein, we explored the association between ABO blood group phenotypes and COVID-19 susceptibility and severity in a Saudi Arabian cohort.

**Methods:**

In this retrospective cohort study, we performed ABO typing on a total of 373 Saudi patients infected with SARS-CoV-2 and conducted association analysis between ABO blood group phenotype and COVID-19 infection severity. We then performed gender-stratified analysis by dividing the participating patients into two groups by gender, and classified them according to age.

**Results:**

The frequencies of blood group phenotypes A, B, AB and O were 27.3, 23.6, 5.4 and 43.7%, respectively. We found that blood group phenotype O was associated with a lower risk of testing positive for COVID-19 infection (OR 0.76 95% CI 0.62–0.95, *p* = 0.0113), while blood group phenotype B was associated with higher odds of testing positive (OR 1.51 95% CI 1.17–1.93, *p* = 0.0009). However, blood group phenotype B was associated with increased risk in the mild and moderate group but not the severe COVID-19 infection group. Blood group phenotype O was protective in all severity groups.

**Conclusion:**

Our findings provide evidence that blood group phenotype B is a risk for COVID-19 disease while blood group phenotype O is protective from COVID-19 infection. However, further studies are necessary to validate these associations in a larger sample size and among individuals of different ethnic groups.

## Introduction

The rapid global spread of severe acute respiratory syndrome coronavirus 2 (SARS-CoV-2) and the resulting coronavirus disease 2019 (COVID-19) has caused drastic morbidity and mortality, igniting an unprecedented effort from scientists and clinicians to rapidly unravel the pathogenesis of disease [1]. COVID-19 is clinically characterized by a multitude of symptoms that vary in severity [2]. The most common symptoms are: fever, cough, fatigue, headache, shortness of breath, conjunctivitis and gastrointestinal problems [3]. There is remarkable variability in viral susceptibility and disease severity caused by SARS-CoV-2 infection, while certain individuals exhibit almost no symptoms, others experience acute respiratory distress syndrome, septic shock, and even death [4, 5].

The ABO blood group system, which is widely used in clinical practice, has been shown in previous studies to confer differential viral susceptibility and disease severity caused by viruses, including SARS-CoV-1 [6]. Indeed, blood groups can play a direct role in infection by serving as receptors or co-receptors for viruses [7]. As with SARS-CoV-1, preliminary evidence suggests a potential correlation between blood group antigens and increased susceptibility to or amplified severity of COVID-19 disease [8–11]. Notably, a higher risk of severe COVID-19 was observed among individuals with blood group A [12], while blood group O was a protective factor [13].

Given that SARS-CoV-2 infection is heterogeneous in clinical presentation and disease course, it is imperative that immunological biomarkers are further elucidated to better define therapeutic strategies and treatment options for COVID-19. Differences in ABO blood group antigen expression may help explain discrepancies of COVID-19 spread, severity and mortality. Additionally, insights on how ABO influences the response to COVID-19 may help identify populations with increased risk and create targeted public health policies. Hence, we performed ABO typing on 373 Saudi patients infected with SARS-CoV-2 to explore the relationship between ABO blood group phenotypes and COVID-19 susceptibility and severity. We further classified the cohort according to gender and age.

## Methods

### Ethics Statement and Patient Recruitment

Ethical approval for this study and all experimental protocols was obtained from the Institutional Review Board (IRB) at King Abdullah International Medical Research Center (KAIMRC), Ministry of National Guard—Health Affairs (MNGHA) in Riyadh and site-specific approval was obtained from all participating centers. Written informed consent for clinical genotyping and participation in this study was obtained from all patients or their guardians upon recruitment. This retrospective cohort study recruited a total of 373 patients with confirmed COVID-19, which was defined as a positive SARS-CoV-2 viral RNA polymerase chain reaction (PCR) test from nasopharyngeal swabs or lower respiratory tract samples including endotracheal aspirate, bronchoalveolar lavage fluid or sputum. All patients were recruited from King Abdulaziz Medical City (KAMC) in Riyadh and Ministry of Health Quarantine Facility in Makkah and recruitment was conducted between May 2020 and August 2020.

### Data Collection

Detailed demographic information, clinical data and laboratory findings were collected from all recruited patients and entered into the Saudi Human Genome Program repository and a secure REDCap electronic data capture tool hosted at KAIMRC. Demographics composed of different factors, such as gender, age, nationality and comorbidities. Clinical data comprised vital signs and disease manifestations including fever, cough, dyspnea, diarrhea and chest pain.

### Patient Phenotype

The cohort included 27 patients whose disease severity was categorized as asymptomatic; 88 patients whose disease severity was categorized as mild, which was defined as patients requiring only self-isolation with mild symptoms, such as fever, headache and cough; 88 patients whose disease severity was categorized as moderate, which was defined as patients requiring hospital admission and evidence of lower respiratory disease during clinical assessment or imaging; and 170 patients whose disease severity was categorized as severe, which was defined as patients requiring ICU admission and/or invasive mechanical ventilation. The asymptomatic and mild group were merged and labeled “mild” for simplicity.

### Sample Collection, DNA Analysis and ABO Typing

Blood samples were collected in EDTA tubes from all 373 patients infected with SARS-CoV-2 and human genomic DNA was extracted from peripheral blood using the Gentra Puregene Blood Kit according to the manufacturer’s instructions. The yield of the DNA was then quantified using NanoDropTM spectrophotometer using standard procedures before genotyping. ABO antigens of all 373 patients were typed by next generation sequencing (NGS) using the Illumina NGS Platform at the laboratories of Histogenetics (Ossining, NY). Briefly, amplification primers were designed for exon 2, exon 6 and exon 7, one amplicon each for exon 2 and 6, and 3 amplicons for exon 7. ABO exon 7 is 698 base pairs long, 3 overlapping amplicons were developed to phase the allelic sequences. When phasing was not possible with NGS data, PacBio 1 kb amplicon was used for phasing exon 7. When discrepancies were found between sequence-based genotyping and serological blood ABO phenotyping, the entire exons (total 9 exons) were sequenced. Blood groups were recorded as phenotype.

### Statistical Analysis

The STATA software, version 16.0 (StataCorp, College Station, TX, USA) was used for statistical analysis. Characteristics of the study population were examined and stratified by ABO blood group phenotype and disease severity using descriptive statistics for age and gender. Chi-square test was employed to assess the statistical significance of differences in proportions of blood group phenotypes between the two groups. Age was expressed as mean values ± SD. Associations between different blood group phenotypes and disease severity were analyzed using the Odds Ratio (OR) with 95% confidence interval (CI). *p* value of less than 0.05 was taken as significant.

## Results

The present study consisted of a total of 373 patients with COVID-19-positive PCR. Of the 373 patients, 218 (58.4%) were males and 155 (41.6%) were females. The age range of the patient cohort was 15–104 years with a mean age of 53.49 (Table 1). The frequencies of blood group phenotypes A, B, AB and O were 27.3, 23.6, 5.4 and 43.7%, respectively. The degree of disease severity increased with age. The analysis showed no statistical significance between gender and severity, however, severe cases were higher in the male gender category while mild cases were higher in females.

**Table 1:** Demographics of the COVID-19 patient cohort

Disease severity	Mean age (years)	SD
Mild	37.00	15.89
Moderate	57.02	15.40
Severe	62.82	14.44
Total	53.49	18.83

*SD* standard deviation

Table 2 illustrates the distribution of patients’ gender and disease severity in relation to age. In both gender categories, age increased with disease severity. However, there was no significant difference in the distribution of patients in the gender versus disease severity.

**Table 2:** Association between gender and COVID-19 disease severity in relation to mean age and SD

Severity	Female	Male	Total
Mild			
Mean	38.02	36.11	36.99
SD	16.02	15.85	15.89
No. (%)	53 (34.19)	62 (28.44)	115
Moderate			
Mean	57.35	56.78	57.02
SD	17.38	13.97	15.40
No. (%)	37 (23.87)	51 (23.39)	88
Severe			
Mean	64.25	61.93	62.82
SD	13.43	15.03	14.44
No. (%)	65 (41.94)	105 (48.17)	170
Total			
Mean	53.63	53.39	53.49
SD	19.16	18.63	18.83
No. (%)	155 (100%)	218 (100%)	373

*No.* number, *SD* standard deviation

Chi^2^(2) for severity and gender distribution = 1.75, *p* = 0.42

Table 3 illustrates the association between age, blood group phenotype and disease severity. In all blood group phenotypes, age increased with disease severity. However, there was no significant difference in the distribution of patients in the severity versus blood group.

**Table 3:** Association between age blood group phenotype and disease severity

Severity	A	B	AB	O	Total
Mild					
Mean age	32.50	35.96	41.83	39.63	36.99
SD	10.12	14.63	17.34	18.65	15.88
*N* (%)	30 (26.09)	28 (24.35)	6 (5.22)	51 (44.35)	115 (100.00)
Moderate					
Mean age	56.29	56.70	56.17	57.76	57.02
SD	13.46	14.89	17.74	16.86	15.40
*N* (%)	21 (23.86)	23 (26.14)	6 (6.82)	38 (43.18)	88 (100.00)
Severe					
Mean age	61.57	62.85	65.25	63.39	62.82
SD	12.81	15.47	13.35	15.27	14.44
*N* (%)	51 (30.00)	37 (21.76)	8 (4.71)	74 (43.53)	170 (100.00)

Chi^2^ = 1.91, *p* = 0.93

**Fig. 1:**
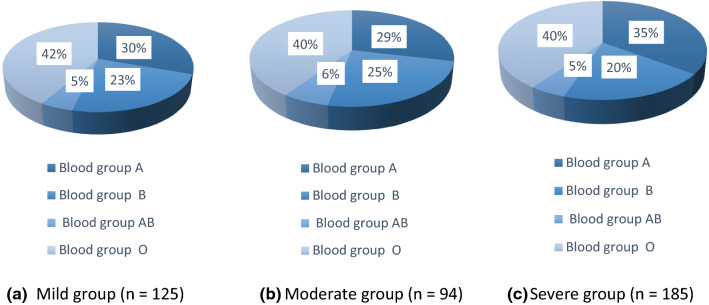
Blood group distribution of the **a** mild COVID-19 patient cohort **b** moderate COVID-19 patient cohort and **c** severe COVID-19 patient cohort

Figure 1 illustrates the blood group distribution in each COVID-19 patient category. In the mild group, the frequencies of blood group phenotypes O, A, B and AB were 42, 30, 23 and 5%, respectively. In the moderate cases, the frequencies of O, A, B and AB were 40, 29, 25 and 6%, respectively. In the severe group, the frequencies of blood group phenotypes O, A, B and AB were 40, 35, 20 and 5%, respectively.

Comparing the COVID-19-infected patients to the general population, we found an increased risk for blood group phenotypes B and AB and a significant decreased risk in blood group phenotype O. Table 4 shows that blood group phenotype B carried a risk for COVID-19 disease (OR 1.51, 95% CI 1.17–1.93, *p* = 0.0009). This association was seen mainly in mild disease (OR 1.57, 95% CI 0.98–2.44, *p* = 0.038) and moderate disease (OR 1.73, 95% CI 1.02–2.83, *p* = 0.023). However, this association did not reach significance for the severe disease (OR 1.36, 95% CI 0.91–1.98, *p* = 0.12). At the same time, blood group phenotype O appears to be protective for COVID-19 disease (OR 0.76, 95% CI 0.62–0.95, *p* = 0.011). This was the case for all disease severity categories, although the significance was not achieved. In addition, only in the total group, blood group AB carried a risk for COVID-19 disease (OR 1.8, 95% CI 1.11–3.00, *p* = 0.0072).

**Table 4:** Influence of disease severity on the ABO blood group phenotype distribution in patients with COVID-19

Controls *N* = 9939				
Blood group	A	B	AB	O
* N* (%)	2840 (28.57%)	1690 (17.00%)	402 (4.04%)	5007 (50.38%)
COVID-19 *N* = 373				
* N* (%)	102 (27.35%)	88 (23.59%)	20 (5.36%)	163 (43.70%)
χ^2^	0.27	10.94	7.22	6.41
* p*	0.61	0.0009	0.0072	0.0113
OR	0.94	1.51	1.88	0.76
95% CI	0.74–1.19	1.17–1.93	1.11–3.00	0.62- 0.95
Mild *N* = 115				
* N* (%)	30 (26.09%)	28 (24.35%)	6 (5.22%)	51 (44.35%)
χ^2^	0.34	4.33	0.40	1.65
* p*	0.56	0.0375	0.5263	0.1985
OR	0.88	1.57	1.31	0.79
95% CI	0.57–1.36	0.98–2.44	0.47–2.96	0.53–1.16
Moderate *N* = 88				
* N* (%)	21 (23.86%)	23 (26.14%)	6 (6.82%)	38 (43.18%)
χ^2^	0.95	5.14	1.72	1.81
* p*	0.3299	0.0234	0.1898	0.1789
OR	0.78	1.73	1.74	0.75
95% CI	0.46–1.30	1.02–2.83	0.615–0.98	0.477–1.167
Severe *N* = 170				
* N* (%)	51 (30.00%)	37 (21.76%)	8 (4.71%)	74 (43.53%)
χ^2^	0.17	2.67	0.19	3.14
* p*	0.6834	0.102	0.6648	0.0766
OR	1.07	1.36	1.17	0.76
95% CI	0.75–1.50	0.91–1.98	0.49–2.39	0.55–1.04

## Discussion

Immense inter-individual susceptibility to SARS-CoV-2 infection and clinical variability in the course of COVID-19 disease has been observed, ranging from silent or benign infection to rapid progression to respiratory failure [2]. Previous work has demonstrated that ABO allele frequencies and distributions of the ABO gene can be appropriate indices for investigating vulnerability to certain infections or disease severity following infections, including SARS-CoV-1 [6]. Correspondingly, recent studies have identified correlations between ABO blood group phenotypes and risk of SARS-CoV-2 infection, as well as susceptibility to severe ICU-requiring COVID-19 disease [14–20]. Hence, we sought to explore these trends among the population in Saudi Arabia. Herein, we performed ABO typing on 373 Saudi patients with confirmed SARS-CoV-2 infection and conducted association analysis between ABO blood group phenotypes and COVID-19.

Overall, we found no significant associations between blood group phenotype and COVID-19 severity. However, we found evidence for associations between blood group phenotype and risk of infection. Several articles were similar in reporting that risk was increased among type A individuals and decreased among O type individuals. However, some articles reported contradicting observations, suggesting that blood group phenotype B individuals are more susceptible to SARS-CoV-2 infection instead of type A. We found that individuals with blood group phenotypes B and AB were at higher odds of testing positive for COVID-19, while type O individuals were less likely to test positive. In Saudi Arabia, the distribution of blood group phenotypes O, A, B, and AB was 50, 29, 17 and 4%, respectively [21]. This suggests that it is likely that the rate of infection is highest in O type individuals. Meanwhile, we found that individuals with blood group phenotypes B and AB were at higher odds of testing positive for COVID-19, while type O individuals were less likely to test positive. Our findings are mostly consistent with the findings reported by Latz et al. who showed that patients with blood group phenotypes B and AB were more likely to test positive while blood group phenotype O was less likely to test positive for COVID-19 [14]. Intriguingly, another study from Saudi Arabia reported similar observations, suggesting that patients with AB blood group have higher susceptibility while patients with O blood group have lower susceptibility to COVID-19 infection [15]. Two studies that were performed in populations closely related to the Saudi population found similar associations. Almadhi and colleagues found a significantly increased risk with blood group phenotype B instead of A in Bahrain [16]. Moreover, Al-Youha et al. detected a lower prevalence of blood group phenotype O in COVID-19 patients and a higher prevalence of B and AB [17]. However, a recent study from Saudi Arabia reported contradicting results, stating that blood group phenotype O individuals are at increased risk for COVID-19, while AB individuals are at decreased risk [18]. This discrepancy may indicate that hidden confounders may be inherent in the tested populations. Also, the variation in results could be attributed to the modest size and suggests that larger sample sizes are needed to study these associations more precisely in Saudi Arabia.

Interestingly, although the variability in the data was mainly in blood group phenotypes A and B, almost all mentioned studies appear to correlate blood group phenotype O with a lower risk for the disease. This is consistent with an association discovered for SARS-CoV-1, in which blood group phenotype O was less common in infected individuals [6]. We found that blood group phenotype B was significantly higher in COVID-19 patients, but the severity was not associated as we have seen a 57% higher risk in mild patients and a 73% higher risk in moderate infection. However, the severe patients only had a 36% higher risk. Intriguingly, this correlates with an association discovered by Ziets et al. in which blood group phenotype B had increased risk of intubation but decreased risk of death [19].

Understanding the role of ABO blood group phenotype in impacting susceptibility and severity of COVID-19 offers the opportunity to gain novel insights into disease pathogenesis, risk stratification and response to therapy. To date, this is the third study that investigates the relationship between ABO and COVID-19 in Saudi Arabia. Our study adds to the growing body of literature around the role of ABO in COVID-19 and findings from our study provide further evidence for several associations that have been reported elsewhere. Nevertheless, these findings should not be taken as conclusive due to certain limitations. First, our study is focused solely on ABO and overlooks the possibilities that other confounding factors may affect the response to COVID-19. Second, our study is confined to the Saudi population. The ABO gene possesses a high degree of polymorphisms and ABO blood group phenotypes have considerably different distributions across populations [22]. Thus, one cannot necessarily assert that the findings presented in this study would apply to other populations or ethnic groups. Finally, the sample size was relatively small and the prevalence of each blood group phenotype is not uniform, with a particularly low sample size in the AB blood group phenotype. Additional studies are necessary to validate these findings in a larger sample size and among individuals of different ethnicities.

In conclusion, our findings provide evidence that blood group B might be a risk factor for COVID disease, while blood group O could be protective from COVID-19 infection in a uniquely Saudi cohort. Nevertheless, the mechanistic link between blood group phenotypes and COVID-19 infection remains elusive. Thus, further interrogation of current findings, both as to their usefulness in risk stratification of patients with COVID-19 and toward a mechanistic understanding of the underlying pathophysiology, is warranted.

## Data Availability

The authors confirm that the data supporting the findings of this study are available within the article and its supplementary materials.
